# Supplementary material for: Novel Spirocyclic Dimer, SpiD3, Targets Chronic Lymphocytic Leukemia Survival Pathways with Potent Preclinical Effects
Source: Cancer Res Commun. 2024 May 22;4(5):1328–43. doi: 10.1158/2767-9764.CRC-24-0071 (PMC11110724; doi:10.1158/2767-9764.CRC-24-0071)
Supplement: Figure S1 — shows the WGCNA-identified modules in SpiD3-treated OSU-CLL cells. [file crc-24-0071-s02.pdf]

Figure S1: WGCNA-identified modules in SpiD3-treated OSU-CLL cells

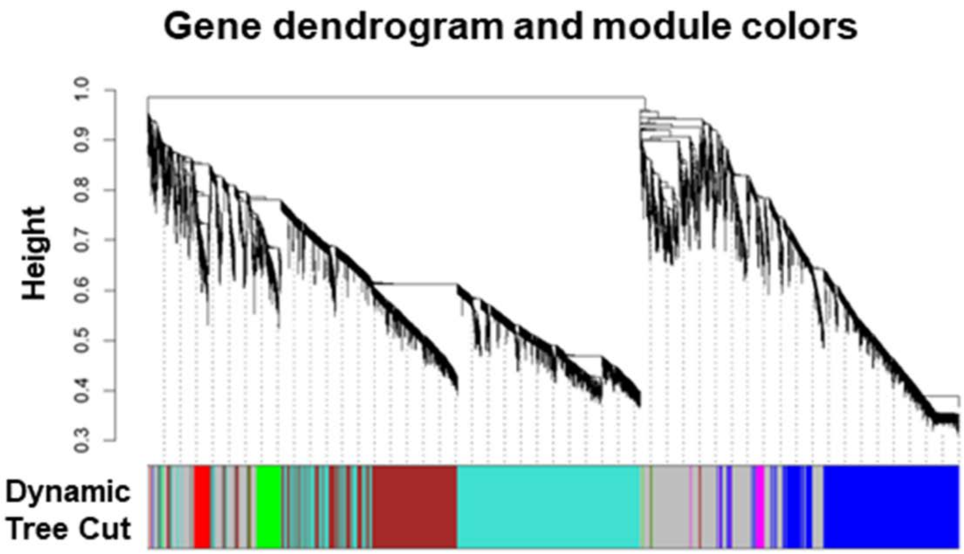

Figure S1. WGCNA-identified modules in SpiD3-treated OSU-CLL cells.
OSU-CLL cells treated with vehicle or SpiD3 (1, 2 μM) for 4 h were subject to RNA-sequencing analysis and weighted gene co-expression network analysis (WGCNA). Gene dendrogram and colors of WGCNA-identified modules for the top 500 differentially expressed genes (P = 0.05) in SpiD3-treated cells are shown.
