# Supplementary material for: Novel Spirocyclic Dimer, SpiD3, Targets Chronic Lymphocytic Leukemia Survival Pathways with Potent Preclinical Effects
Source: Cancer Res Commun. 2024 May 22;4(5):1328–43. doi: 10.1158/2767-9764.CRC-24-0071 (PMC11110724; doi:10.1158/2767-9764.CRC-24-0071)
Supplement: Figure S2 — shows the RNA-sequencing analysis of SpiD3-treated OSU-CLL cells. [file crc-24-0071-s03.pdf]

Figure S2. RNA-sequencing analysis of SpiD3-treated OSU-CLL cells.

(A)

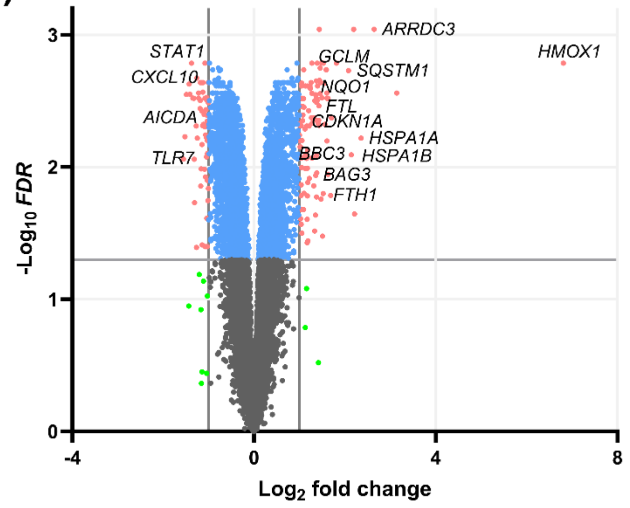

(B)

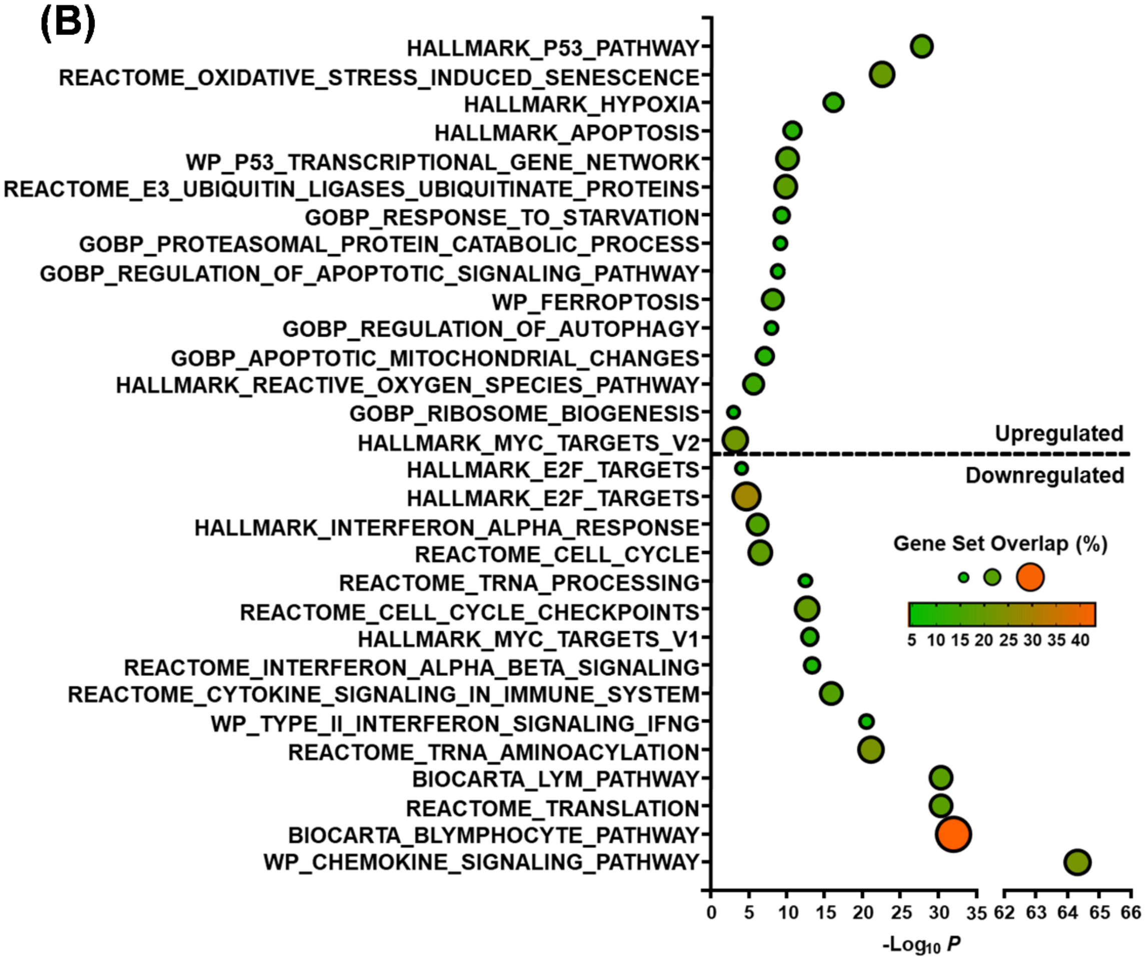

Figure S2. RNA-sequencing analysis of SpiD3-treated OSU-CLL cells.

(A) Volcano plot of differentially expressed genes (DEGs) in SpiD3-treated OSU-CLL cells (1 μM, 4 h) with select disease-relevant genes labeled. Only genes that meet both the statistical significance (*FDR* < 0.05) and fold-change ($|Log_2 FC| > 1$) were used for downstream analysis (red). Genes that meet only statistical significance (blue), only fold-change (green), or neither threshold (grey) are shown for comparison. **(B)** Gene set enrichment analysis using the top 500 DEGs (*P* < 0.05) following treatment with 1 μM SpiD3 (4 h, n = 3 independent RNA-seq experiments).
