# Supplementary material for: Novel Spirocyclic Dimer, SpiD3, Targets Chronic Lymphocytic Leukemia Survival Pathways with Potent Preclinical Effects
Source: Cancer Res Commun. 2024 May 22;4(5):1328–43. doi: 10.1158/2767-9764.CRC-24-0071 (PMC11110724; doi:10.1158/2767-9764.CRC-24-0071)
Supplement: Figure S3 — shows proteomic analysis of SpiD3-treated CLL cells using TMT-labeling. [file crc-24-0071-s04.pdf]

Figure S3. Proteomic analysis of SpiD3-treated CLL cells using TMT-labeling.

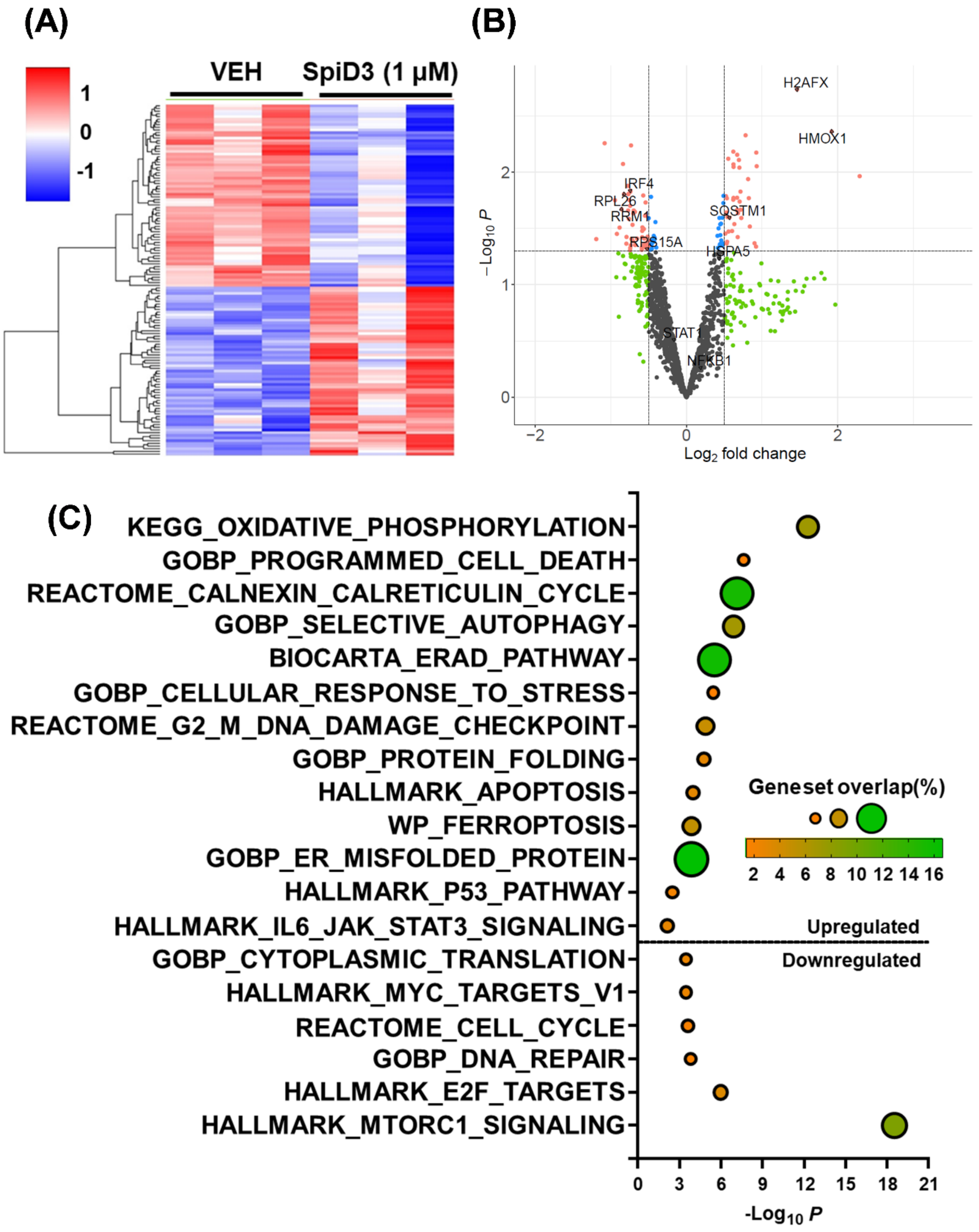

Figure S3. Proteomic analysis of SpiD3-treated CLL cells using TMT-labeling.

(A) Hierarchical clustering of differentially expressed proteins (n = 131, *P* < 0.05) in OSU-CLL cells treated with DMSO vehicle (VEH) or SpiD3 (1 μM) for 24 h (n = 3 independent experiments). **(B)** Volcano plot of differentially expressed proteins in SpiD3-treated OSU-CLL cells with select disease-relevant proteins labeled. Only proteins that meet both the statistical significance (*P* < 0.05) and fold-change ($|Log_2 FC| > 1$) were used for downstream analysis (red). Genes that meet only statistical significance (blue), only fold-change (green), or neither threshold (grey) are shown for comparison. **(C)** Gene set enrichment analysis of the differentially expressed proteins following SpiD3 treatment.
