# Supplementary material for: Novel Spirocyclic Dimer, SpiD3, Targets Chronic Lymphocytic Leukemia Survival Pathways with Potent Preclinical Effects
Source: Cancer Res Commun. 2024 May 22;4(5):1328–43. doi: 10.1158/2767-9764.CRC-24-0071 (PMC11110724; doi:10.1158/2767-9764.CRC-24-0071)
Supplement: Figure S4 — shows SpiD3 inhibits CLL chemotaxis and induces transcription of UPR genes. [file crc-24-0071-s05.pdf]

Figure S4. SpiD3 inhibits CLL chemotaxis and induces transcription of UPR genes.

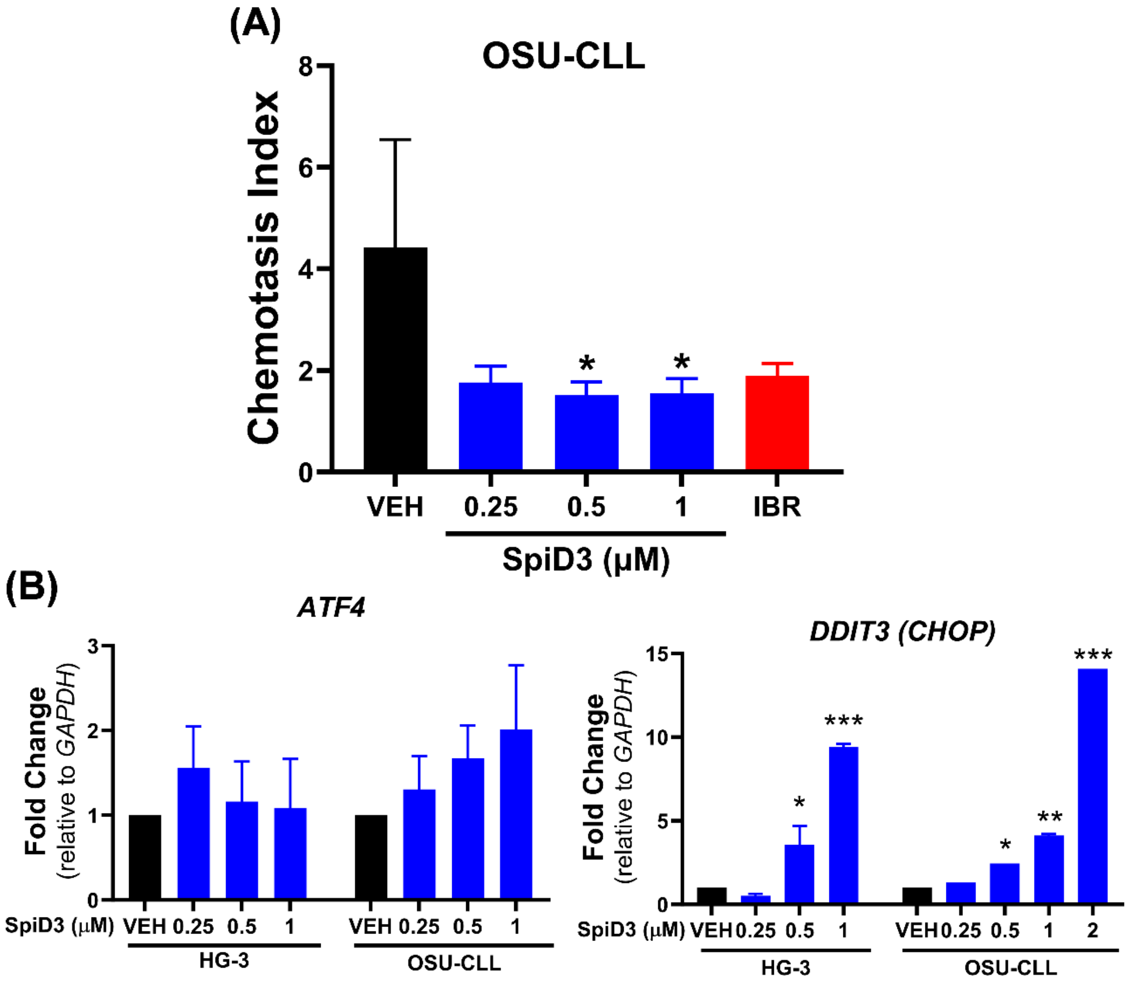

Figure S4. SpiD3 inhibits CLL chemotaxis and induces transcription of UPR genes.

(A) OSU-CLL cells were pre-treated with SpiD3 (0.25-1 µM), ibrutinib (IBR, 1 µM), or equivalent DMSO vehicle (VEH) for 1 h and allowed to migrate for 6 h through trans-well inserts toward 200 ng/mL CXCL-12. The chemotaxis index represents the number of cells that migrated toward the indicated chemokine divided by the number of cells that migrated with no chemokine present for each treatment condition (n = 3 independent experiments). **(B)** Quantitative real-time PCR analysis of *ATF4* and *DDIT3 (CHOP)* in HG-3 and OSU-CLL cells treated with equivalent VEH or SpiD3 (0.25-2 µM) for 4 h (n = 2-3 independent experiments/cell line). Transcript expression is normalized to the housekeeping gene (*GAPDH*). Data are shown as fold change to VEH (mean ± SEM). Asterisks denote significance *vs.* VEH: *P < 0.05, **P < 0.01, ***P < 0.001.
