# Supplementary material for: Novel Spirocyclic Dimer, SpiD3, Targets Chronic Lymphocytic Leukemia Survival Pathways with Potent Preclinical Effects
Source: Cancer Res Commun. 2024 May 22;4(5):1328–43. doi: 10.1158/2767-9764.CRC-24-0071 (PMC11110724; doi:10.1158/2767-9764.CRC-24-0071)
Supplement: Figure S5 — shows SpiD3 inhibits cap-dependent protein translation in CLL cells. [file crc-24-0071-s06.pdf]

**Figure S5. SpiD3 inhibits cap-dependent protein translation in CLL cells.**

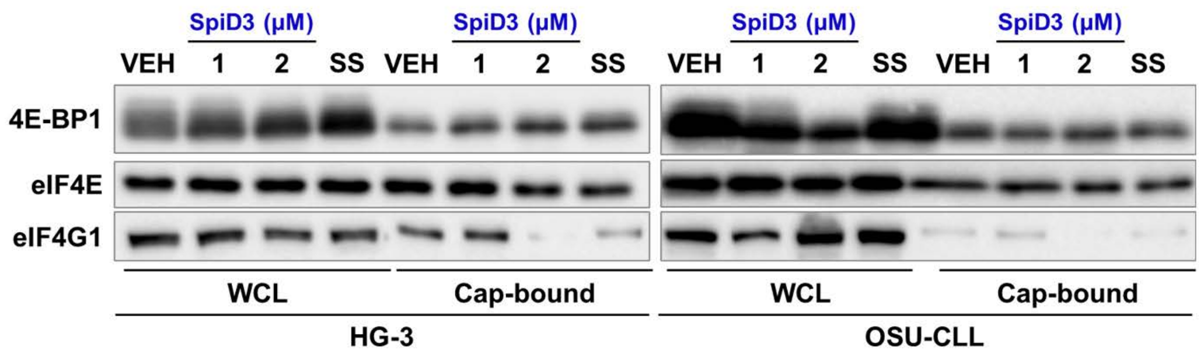


**Figure S5. SpiD3 inhibits cap-dependent protein translation in CLL cells.**
HG-3 and OSU-CLL cells were treated with VEH, SpiD3 (1, 2 μM), or cultured in serum-deprived media (SS) for 8 h. Cell lysates were incubated with 7-methyl-GTP-Sepharose resin (Cap-bound). Eluted cap-bound proteins and whole cell lysates (WCL) were analyzed via immunoblot for 4E-BP1, eIF4E and eIF4G1 (n = 3 independent experiments).
