# Supplementary material for: Novel Spirocyclic Dimer, SpiD3, Targets Chronic Lymphocytic Leukemia Survival Pathways with Potent Preclinical Effects
Source: Cancer Res Commun. 2024 May 22;4(5):1328–43. doi: 10.1158/2767-9764.CRC-24-0071 (PMC11110724; doi:10.1158/2767-9764.CRC-24-0071)
Supplement: Figure S6 — shows SpiD3 inhibits NF-κB activity and diminishes CD40L-induced survival signaling in CLL. [file crc-24-0071-s07.pdf]

Figure S6. SpiD3 inhibits NF-κB activity and diminishes CD40L-induced survival signaling in CLL.

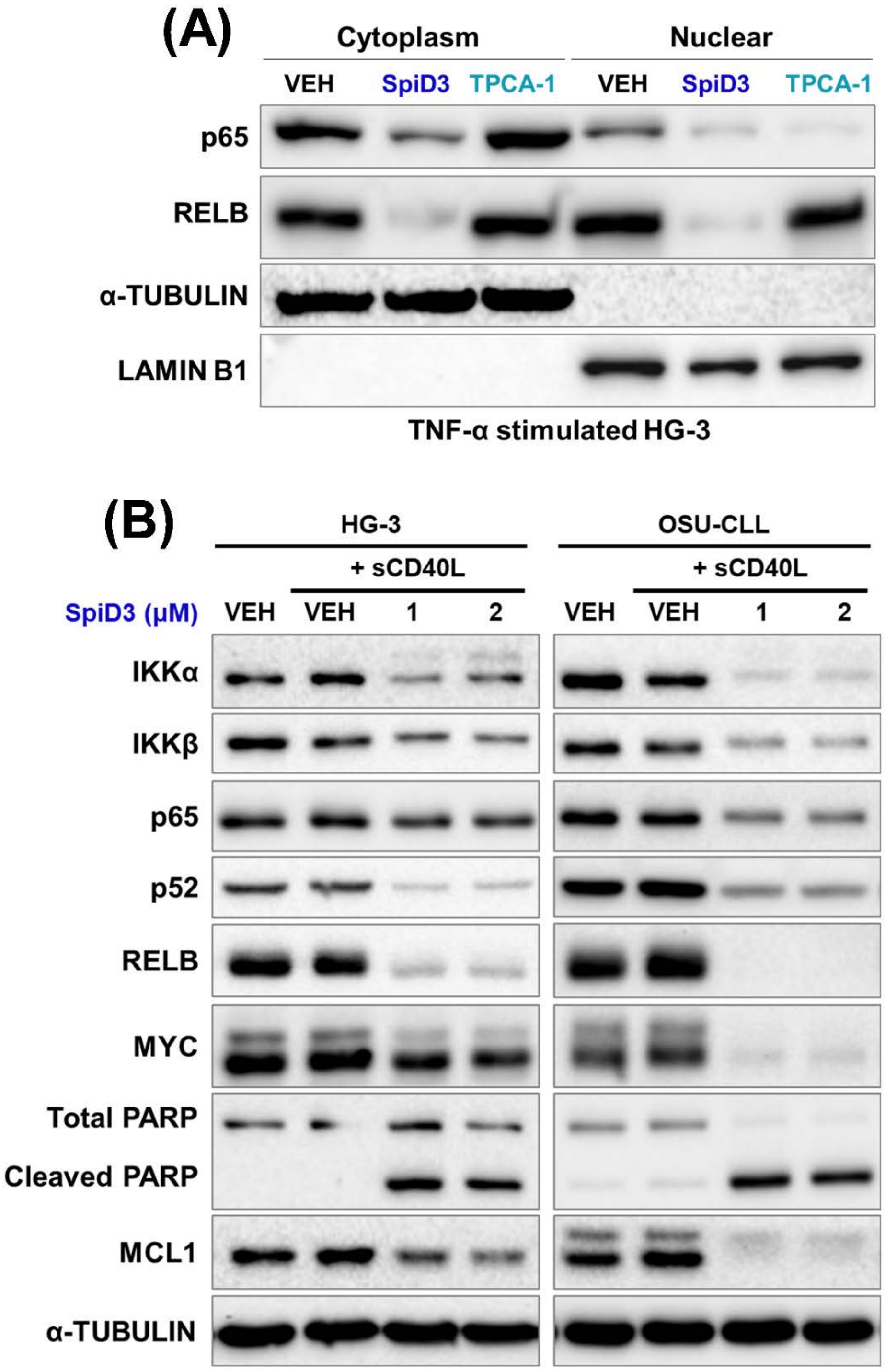

Figure S6. SpiD3 inhibits NF-κB activity and diminishes CD40L-induced survival signaling in CLL.

(A) HG-3 cells were treated with SpiD3 (2 μM), TPCA-1 (10 μM), or equivalent DMSO vehicle (VEH) for 4 h and stimulated with TNF-α (20 ng/mL) during the last 15 min of treatment (n = 4 independent experiments). Cytoplasmic and nuclear fractions were subjected to immunoblotting probing for p65 and RELB. α-TUBULIN served as the cytoplasmic fraction loading control and LAMIN B1 served as the nuclear fraction loading control. **(B)** Immunoblot analysis of the indicated proteins in whole cell lysates of HG-3 and OSU-CLL cells treated with SpiD3 (1, 2 μM) for 4 h and co-currently stimulated with sCD40L (500 ng/mL). α-TUBULIN served as the loading control (n = 4 independent experiments/cell line).
