# Supplementary material for: Novel Spirocyclic Dimer, SpiD3, Targets Chronic Lymphocytic Leukemia Survival Pathways with Potent Preclinical Effects
Source: Cancer Res Commun. 2024 May 22;4(5):1328–43. doi: 10.1158/2767-9764.CRC-24-0071 (PMC11110724; doi:10.1158/2767-9764.CRC-24-0071)
Supplement: Figure S7 — shows SpiD3 spares healthy stromal and lymphoid cells. [file crc-24-0071-s08.pdf]

Figure S7. SpiD3 spares healthy stromal and lymphoid cells.

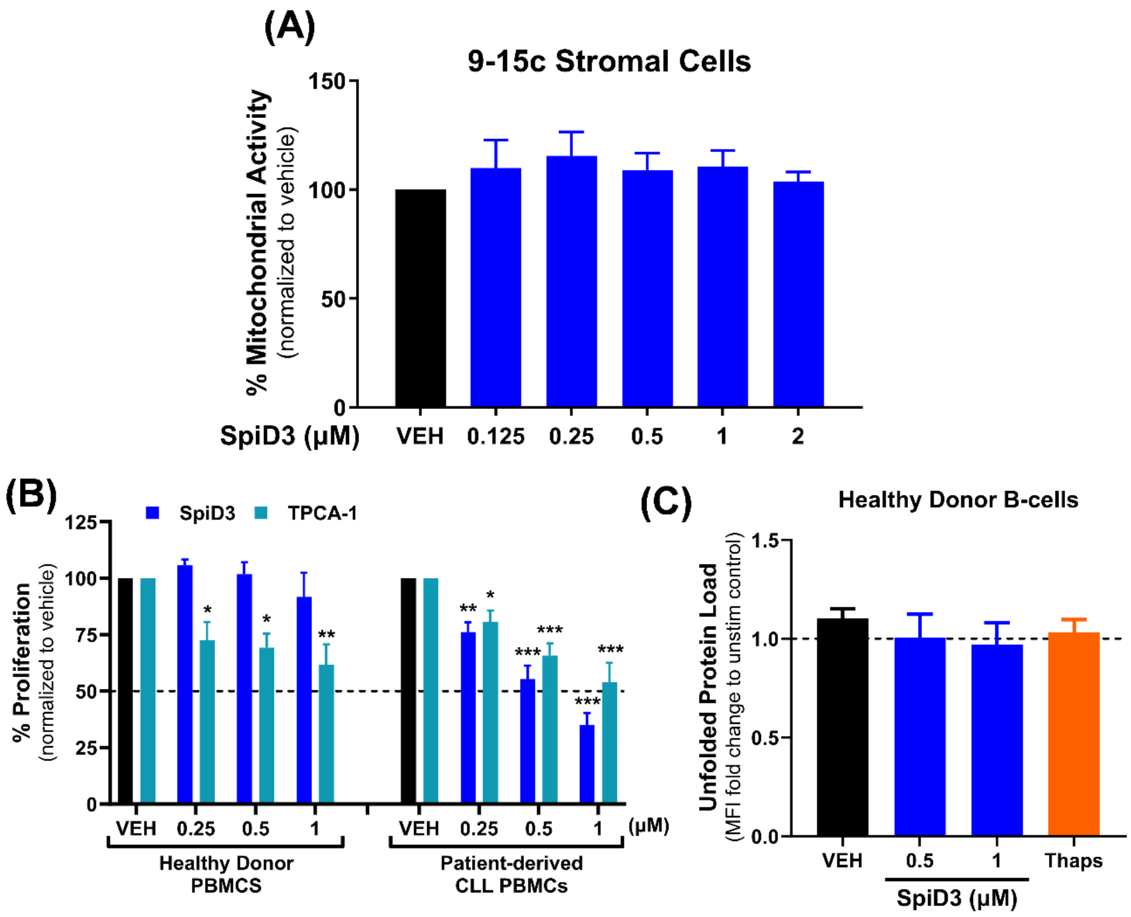

Figure S7. SpiD3 spares healthy stromal and lymphoid cells.

(A) 9-15c bone marrow-derived stroma cells were treated with SpiD3 (48 h). Mitochondrial activity was assessed via MTS assay and results are given as % mitochondrial activity normalized to vehicle (n = 4 independent experiments). **(B)** Healthy donor PBMCs (n = 5) or patient-derived CLL PBMCs (n = 8; > 92% CLL B-cells) were treated with the indicated amounts of SpiD3 or TPCA-1 with co-current CpG ODN 2006 stimulation (CpG; 3.2 μM) for 48 h. Proliferation was assessed via MTS assay and results are given as % proliferation normalized to the CpG-stimulated control. Data are shown as mean ± SEM. Asterisks denote significance *vs*. VEH: **P* < 0.05, ***P* < 0.01, ****P* < 0.001. **(C)** UPR induction in healthy donor B-cells (n = 7) was evaluated following a 24 h ex vivo treatment with SpiD3 or thapsigargin (Thaps; 1 μM) under co-current CpG stimulation (3.2 μM) via incubation with TPE-NMI dye. Data are represented as fold change in TPE-NMI median fluorescence intensity (MFI) compared to the unstimulated control (dashed line).
