# Supplementary material for: Novel Spirocyclic Dimer, SpiD3, Targets Chronic Lymphocytic Leukemia Survival Pathways with Potent Preclinical Effects
Source: Cancer Res Commun. 2024 May 22;4(5):1328–43. doi: 10.1158/2767-9764.CRC-24-0071 (PMC11110724; doi:10.1158/2767-9764.CRC-24-0071)
Supplement: Figure S8 — shows the studies with SpiD3_AP in CLL. [file crc-24-0071-s09.pdf]

Figure S8. Studies with SpiD3_AP in CLL.

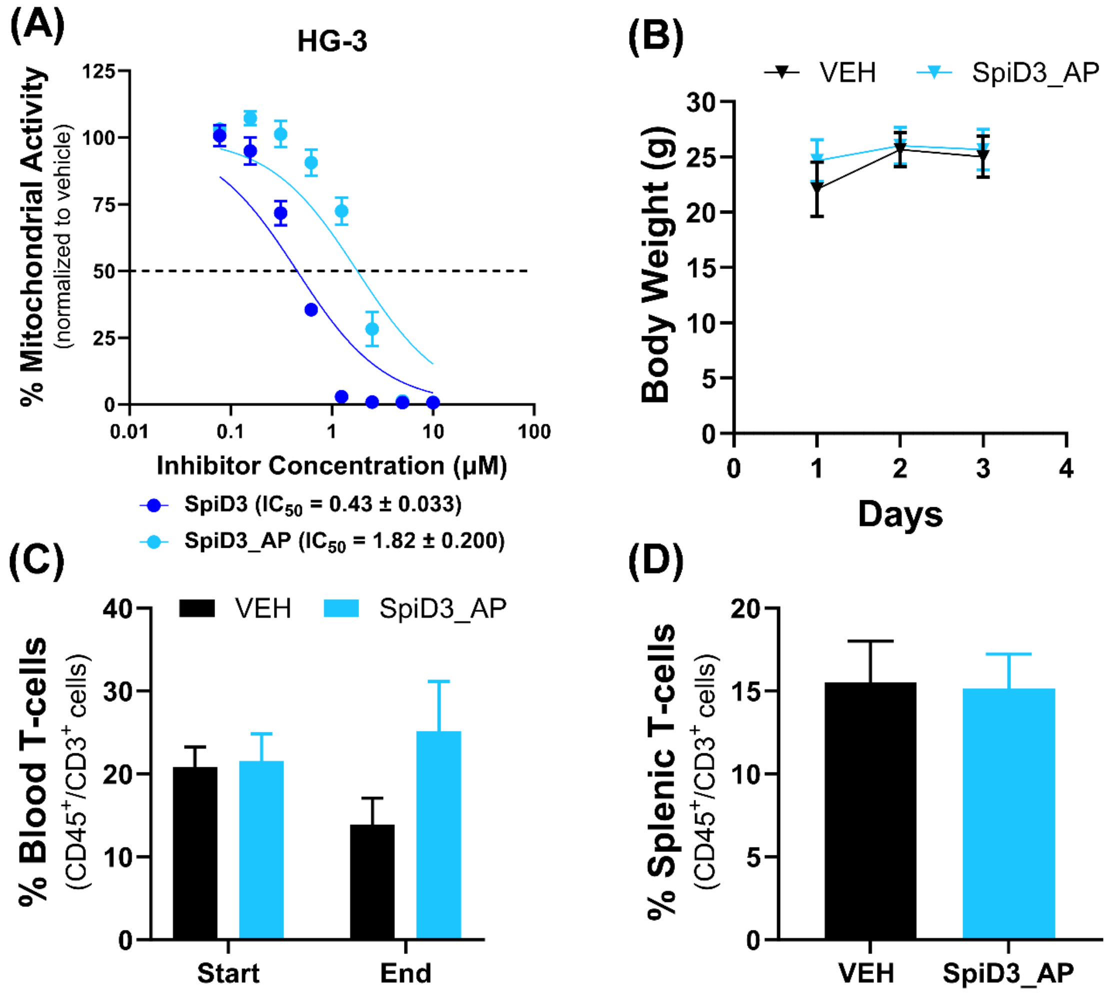

Figure S8. Studies with SpiD3_AP in CLL.

(A) Proliferation of HG-3 CLL cell line was determined by MTS assay following treatment with increasing concentrations of SpiD3 or SpiD3 prodrug (SpiD3_AP) for 72 h (n = 3 independent experiments). Proliferation was assessed via MTS assay and normalized to vehicle. IC_{50} values are shown as mean ± SEM. **(B-D):** Diseased Eμ-TCL1 mice (median age = 10.2 mo) were randomized to receive 10 mg/kg SpiD3_AP or vehicle equivalent (VEH) via intravenously (IV) injection for 3 consecutive days. Equal numbers of male and female mice were used per treatment arm (n = 6 mice/arm). Mouse body weight was monitored throughout the study **(B)**. At study end (~3 h after the last IV injection), mice were sacrificed for tissue harvest. Flow cytometry evaluation of T-cells in blood **(C)** and spleen **(D)**. Data are shown as mean ± SEM.
