# Supplementary material for: Novel Spirocyclic Dimer, SpiD3, Targets Chronic Lymphocytic Leukemia Survival Pathways with Potent Preclinical Effects
Source: Cancer Res Commun. 2024 May 22;4(5):1328–43. doi: 10.1158/2767-9764.CRC-24-0071 (PMC11110724; doi:10.1158/2767-9764.CRC-24-0071)
Supplement: Table S1 — shows the characteristics of the patient-derived CLL samples used in the study. [file crc-24-0071-s10.pdf]

Table S1: Characteristics of the patient-derived CLL samples used in the study

Patient #	IGHV status	Gender	Age	Treatment	FISH Cytogenetics					Karyotype*	Figure(s)
					Deletion (17p)	Deletion (13q)	Deletion (11q)	Deletion (6q)	Trisomy 12		
1	Unmut	M	47	Naïve	Neg	Neg	Neg	Neg	Neg	Normal	Fig. 4B
2	Unmut	M	55	Naïve	Neg	Neg	Pos	Pos	Neg	Complex	Fig. 4B
3	Unmut	F	76	Naïve	Neg	Neg	Pos	Neg	Neg	Normal	Fig. 4A-B
4	Unmut	F	65	Naïve	Neg	Neg	Neg	Neg	Pos	Normal	Fig. 4B
5	NA	M	81	Treated	Neg	Neg	Neg	Neg	Neg	Normal	Fig. 4B
6	NA	F	68	Treated	Neg	Neg	Neg	Neg	Pos	Normal	Fig. 4A
7	Mut	M	94	Treated	Neg	Neg	Neg	Neg	Pos	Normal	Fig. 4A
8	Unmut	M	76	Treated	Pos	Neg	Neg	Neg	Neg	Complex	Fig. 4A
9	Mut	M	47	Naïve	Neg	Neg	Neg	Neg	Pos	Complex	Fig. 4A
10	Unmut	F	62	Naïve	Neg	Neg	Neg	Neg	Pos	Normal	Fig. 4A
11	Unmut	M	76	Treated	Neg	Neg	Neg	Neg	Pos	Normal	Fig. 4A-B
12	Unmut	M	45	Naïve	Neg	Neg	Pos	Neg	Pos	Normal	Fig. 4A
13	NA	M	57	Naïve	Pos	Neg	Neg	Neg	Neg	Complex	Fig. 4A
14	Unmut	M	70	Naïve	Neg	Neg	Neg	Neg	Neg	Normal	Fig. 4A
15	Unmut	M	34	Naïve	Neg	Neg	Neg	Neg	Pos	Normal	Fig. 4A
16	Unmut	F	60	Treated	Neg	Neg	Pos	Neg	Neg	Normal	Fig. 4A
17	Unmut	M	47	Naïve	Neg	Neg	Neg	Neg	Neg	Normal	Fig. 4A
18	Unmut	F	52	Naïve	Neg	Neg	Neg	Neg	Neg	Complex	Fig. 4A
19	Mut	F	66	Treated	Neg	Pos	Neg	Neg	Neg	Normal	Fig. 4B, Fig. 5C-D, Supp. Fig S7B
20	Unmut	F	68	Treated	Neg	Pos	Pos	Neg	Neg	NA	Fig. 4B
21	Mut	F	78	Treated	Neg	Pos	Pos	Neg	Neg	Complex	Fig. 4B
22	Mut	M	80	Naïve	Neg	Pos	Neg	Neg	Neg	NA	Fig. 4C, Fig. 5C-D, Supp. Fig S7B
23	Unmut	F	53	Naïve	Neg	Pos	Neg	Neg	Neg	Normal	Fig. 4B-D
24	NA	M	66	Naïve	NA	NA	NA	NA	NA	NA	Fig. 4D
25	Mut	M	78	Naïve	Neg	Pos	Neg	Neg	Neg	Normal	Fig. 4B
26	Unmut	M	65	Naïve	Neg	Neg	Neg	Neg	Neg	NA	Fig. 4B, Fig. 4D, Fig. 5C-D
27	Unmut	F	65	Naïve	Neg	Pos	Pos	Neg	Neg	NA	Fig. 4B-D, Supp. Fig S7B
28	Mut	M	45	Treated	Neg	Neg	Neg	Neg	Neg	NA	Fig. 4D
29	NA	M	66	Naïve	Neg	Neg	Neg	Neg	Neg	Normal	Fig. 4C, Fig. 4E, Fig. 5C-D, Supp. Fig S7B
30	NA	F	58	Naïve	NA	NA	NA	NA	NA	NA	Fig. 4B-C
31	Unmut	M	45	Treated	Neg	Pos	Neg	Neg	Neg	Normal	Fig. 4B-C
32	Unmut	M	72	Naïve	Neg	Neg	Neg	Neg	Neg	Normal	Fig. 4D
33	Unmut	M	70	Naïve	Pos	Neg	Neg	Neg	Neg	NA	Fig. 4B, Fig. 4E, Supp. Fig S7B
34	Unmut	F	68	Treated	Neg	Neg	Neg	Neg	Neg	Normal	Fig. 4A, Fig. 4C-D, Supp. Fig S7B
35	Unmut	M	68	Naïve	Neg	Neg	Neg	Pos	Neg	NA	Fig. 4B, Fig. 5C-D
36	NA	F	75	Treated	Pos	Pos	Neg	Neg	Neg	Complex	Fig. 4D, Fig. 5C-D
37	Mut	M	50	Naïve	Neg	Pos	Neg	Neg	Neg	Normal	Fig. 4B, Supp. Fig S7B
38	Unmut	M	61	Treated	Neg	Neg	Neg	Neg	Pos	Normal	Fig. 4D, Fig. 5C-D
39	NA	F	82	Treated	Neg	Pos	Neg	Neg	Neg	Normal	Fig. 4B, Supp. Fig S7B
40	Unmut	M	59	Naïve	Neg	Pos	Pos	Neg	Neg	NA	Fig. 4B

IGHV: immunoglobulin heavy-chain variable region gene, Unmut: unmutated *IGHV*, Mut: mutated *IGHV*; M: male, F: female; Neg: negative, Pos: positive; NA: not available, Fig: Figure. *Complex karyotype is defined as >3 cytogenetic abnormalities.
