# Supplementary material for: Novel Spirocyclic Dimer, SpiD3, Targets Chronic Lymphocytic Leukemia Survival Pathways with Potent Preclinical Effects
Source: Cancer Res Commun. 2024 May 22;4(5):1328–43. doi: 10.1158/2767-9764.CRC-24-0071 (PMC11110724; doi:10.1158/2767-9764.CRC-24-0071)
Supplement: Table S2 — is the list of primary antibodies used for immunoblotting assays in the study. [file crc-24-0071-s11.pdf]

Table S2: List of primary antibodies used for immunoblotting assays in the study

Primary Antibodies	Catalog	RRID
**Cell Signaling Technology**		
p4E-BP1 (Ser65)	9456	RRID:AB_823413
4E-BP1	9644	RRID:AB_2097841
p-BTK (Tyr223)	87457	RRID:AB_2800102
BTK	8547	RRID:AB_10950506
CHOP	2895	RRID:AB_2089254
p-eIF2α (Ser51)	3398	RRID:AB_2096481
eIF2α	5324	RRID:AB_10692650
eIF4A	2490	RRID:AB_823487
eIF4E	2067	RRID:AB_2097675
eIF4G	2469	RRID:AB_2096028
p-ERK1/2 (Thr202/Tyr204)	4377	RRID:AB_331775
ERK1/2	4695	RRID:AB_390779
GAPDH	5174	RRID:AB_10622025
γH2A.X (Ser139)	9718	RRID:AB_2118009
HO-1/HMOX1	43966	RRID:AB_2799254
IKKβ	8943	RRID:AB_11024092
IRE1α	3294	RRID:AB_823545
MCL1	5453	RRID:AB_10694494
MYC	5605	RRID:AB_1903938
p21	2947	RRID:AB_823586
p52	4882	RRID:AB_10695537
PARP	9542	RRID:AB_2160739
PDCD4	9535	RRID:AB_2162318
PERK	5683	RRID:AB_10841299
p-PRAS (Thr246)	13175	RRID:AB_2798140
PRAS	2691	RRID:AB_2225033
RELB	10544	RRID:AB_2797727
**Invitrogen**		
LAMIN B1	702972	RRID:AB_2784553
XBP1 (spliced/unspliced)	PA5-27650	RRID:AB_2545126
**Santa Cruz Biotechnology**		
β-ACTIN	sc-47778	RRID:AB_626632
IKKα	sc-52932	RRID:AB_783991
p65	sc-8008	RRID:AB_628017
**Sigma**		
α-TUBULIN	T5201	RRID:AB_609915
**Proteintech**		
ATF4	10835	RRID:AB_2058600
